# Pseudocapsule and pseudocapsule-based extracapsular resection in pituitary neuroendocrine tumors

**DOI:** 10.3389/fendo.2022.1056327

**Published:** 2022-11-18

**Authors:** Xiao Bin Wang, Tian Yi Han, Jian Gong Ma, Cheng He, Li Xue, Xun Zhang, Zhe Bao Wu

**Affiliations:** ^1^ Department Of Neurosurgery, The First Affiliated Hospital of Henan University, Kaifeng, Henan, China; ^2^ Department Of Neurosurgery, Center of Pituitary Tumors, Ruijin Hospital, Shanghai Jiao Tong University School of Medicine, Shanghai, China; ^3^ Neuroendocrine Research Laboratory, Massachusetts General Hospital and Harvard Medical School, Boston, MA, United States

**Keywords:** pituitary neuroendocrine tumors, pseudocapsule, pseudocapsule-based extracapsular resection, intracapsular resection, neuroendoscopy

## Abstract

Since Costello et al. proposed the concept of pseudocapsule of pituitary neuroendocrine tumors (PitNETs) in 1936, many studies have been published on its occurrence, development process, histopathology, and morphology. Pseudocapsule has been proposed as the anatomical interface between PitNETs and normal pituitary gland, therefore the so-called pseudocapsule-based extracapsular resection (ER) technique was developed as an extracapsular surgery method for PitNETs,which differs from the conventional intracapsular resection (IR). In recent years, ER has also been widely used in patients of different tumor types, sizes, and age groups, because the pseudocapsule can be identified more clearly under the endoscopy. Endoscopic transsphenoidal resection for PitNETs has become the preferred surgical method. We reviewed relevant literatures in the past 10 years, showing that ER could achieve better rate of gross total resection (GTR) and biochemical remission, and reduce tumor recurrence than IR, without increasing postoperative complications. Therefore, the pseudocapsule and ER should be valued by neurosurgeons and actively promoted clinically.

## Introduction

Pituitary neuroendocrine tumors (PitNETs) are common benign intracranial tumors, accounting for about 10-15% of intracranial tumors. The incidence of PitNETs seems to have taken the second place among intracranial tumors in recent years, only after meningiomas (1, 2). Based on the tumor size, PitNETs can be classified as microadenomas(<10mm), macroadenomas (≥10mm, <40mm) and giant adenomas (≥40mm). The new WHO classification of PitNETs in 2022 has proposed a detailed histological classification of tumors based on cell lineages, cell types, hormones, and other auxiliary features (3). Somatotropin-, prolactin-, prolactin-somatotropin- and thyrotropin-producing cells belong to the PIT1 cell lineage; corticotrophs belong to the TPIT cell lineage; and gonadotropin producing cells belong to the SF1 cell lineage. Therefore, the new version of PitNETs classification in 2022 contains a total of four categories, including PIT1 lineage, TPIT lineage, SF1 lineage and PitNETs without distinct cell lineage. Typical clinical symptoms of PitNETs are visual/visual field impairment caused by compression of the optic nerves/chiasm; amenorrhea, lactation, acromegaly, centripetal obesity and hyperthyroidism caused by disorders of pituitary hormones; headache and vomiting caused by increased intracranial pressure. PitNETs can affect the fertility and growth, and causing emotion disorders and cognitive dysfunctions in patients. Treatments for PitNETs include surgical removal, pharmacotherapy, radiotherapy, the replacement of pituitary hormones, as well as clinical observation. Surgical removal is the preferred treatment for the most PitNETs. Trans-Nasal Trans-Sphenoidal (TNTS) resection has become the most common approach to PitNETs (4–6).

Since the middle of last century, some scholars have been proposing that there was a layer of membranous anatomical structure between PitNETs and normal pituitary tissue, so called the pseudocapsule. Many detailed studies on its occurrence, development process, histopathology and morphology have been conducted. Meanwhile, ER along anatomical interface has been developed as an extracapsular surgery method of PitNETs (7–13). However, due to the equipmental and technical limitations, the identification of the pseudocapsule remained unclear for quite a while. With the development of neuroendoscopy, better illumination source, high-definition video, closer observation, and local magnification of microscopic structures, the pseudocapsule now can be more clearly distinguished, therefore promoting the pseudocapsule-based extracapsular resection (ER) technique. It has been suggested that ER can largely protect normal pituitary functions while totally resect the tumors, thus greatly improving the tumor resection rate and endocrine remission rate, conserving pituitary functions, and lowering the recurrence rate. Therefore the pseudocapsule and ER need to receive more attentions for further development and wider application.

In this review, we analyzed related publications in the past 10 years, summarized the characteristics of pseudocapsule and the surgical effects of ER in PitNETs by neuroendoscopy or microscopy with neuroendoscopy-assisted transsphenoidal surgery, in order for a comprehensive understanding of the pseudocapsule and the ER of PitNETs for neurosurgeons.

## The origin and evolution of the terminology pseudocapsule

In 1936, Costello et al. discovered at autopsy that there was a histological capsule around the PitNETs (7). It was not a real tumor capsule, but a capsule-like structure formed as the tumor squeezed the surrounding pituitary tissue. Thus, the concept of “pseudocapsule” was first noted. In 1978, Wrightson et al. found that there was a clear boundary between the PitNETs and normal tissues when performing pathological examination of PitNET specimens, indirectly confirming the existence of pseudocapsule (8). In 1994, Faraoud et al. discovered the presence of a transitional zone between the adenoma and the surrounding normal pituitary tissue consisting of several layers of peritumoral cell-cords wrapped by the basement membrane (13). In 2005, Kawamata et al. initially put forward the term “microsurgical pseudocapsule”, applied this concept in the resection of GH-secreting pituitary adenomas, and performed a detailed study of the pseudocapsule histopathology (9). In 2006, Oldfield et al. defined the histological pseudocapsule between PitNETs and the normal pituitary tissues as “adenoma surgical capsule”, and described the formation and development of the pseudocapsule of PitNETs in detail for the first time (10). Furthermore, he and his colleagues described the surgical techniques of “pseudocapsule-based extracapsular resection” in great details, which have been widely recognized and is still in use today. This can be regarded as a milestone in the history of the pseudocapsule of PitNETs.

In recent years, neuroendoscopy has been introduced to assist the resection of PitNETs with different hormone types, sizes, and ages, which has made the identification of pseudocapsules clearer during the operations. To this day, research on the histological origin and characteristics of pseudocapsules continues. Although a unified and standard understanding has not yet been accepted, the term “pseudocapsule” first proposed by Costello have been widely recognized, and the “extracapsular resection” technique by Oldfield is being used by many surgeons and continuously improved in PitNET surgeries.

## The characteristics of the pseudocapsule

### Anatomical characteristics

**Figure 1:**
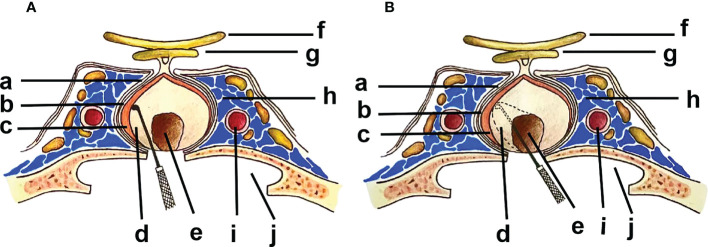
**(a)** Dural Envelope; **(b)** Pituitary Capsule; **(c)** Pituitary Gland; **(d)** Pseudocapsule; **(e)** PitNET; **(f)** Optic Nerve; **(g)** Optic Chiasm; **(h)** Cavernous Sinus; **(i)** ICA (internal carotid artery); **(j)** Sphenoid Sinus. Inward from the medial wall of the cavernous sinus: Dural envelope-Pituitary Capsule-Pituitary gland-Pseudocapsule-PitNET. **(A)**. ER along the outer side of the pseudocapsular anatomical plane. **(B)**. IR along the inner side of the pseudocapsular anatomical plane.

It is currently agreed that there are three layers of the membranous structure around PitNETs (9, 12, 14), from outside to inside (**Figure 1**): 1. The dural envelope, the continuation of the intracranial dura in the sella, which is usually bilayer. However, in most cases, the part involved in the inner wall of the cavernous sinus is extremely thin. 2. Pituitary capsule, which is a dense fibrous tissue capsule between the normal pituitary and the dura mater that completely covers the pituitary, and contains type I-V collagen. 3. Pseudocapsule, which is a compressed reticulin layer formed by the condensed pituitary acini and reticulin located between the adenoma and the normal pituitary gland, resembling to type III collagen. The dural envelope at the medial wall of the cavernous sinus and the pituitary capsule together form a two-layer barrier that prevents the expansion of the PitNETs. The pseudocapsule forms an anatomical interface between the normal pituitary tissues and the PitNETs. However, this anatomical interface is not always present, as the pseudocapsule was only found in about 50% of PitNETs according to the previous studies (11, 15, 16). In recent years, the application of neuroendoscopy provided the clearer field of view, and this anatomical interface is easier to identify during surgery, thus the detection of pseudocapsules has been increased significantly. Some researchers have mentioned that because of the different fluorescence distribution of indocyanine green (ICG) in normal glands, tumors, and dura maters, the pseudocapsule is more likely to be recognized under a fluorescence microscope (17).

### Histopathological characteristics

Early studies did not provide a detailed histological description of the pseudocapsule. Wrightson et al. conducted histopathological study on the tissue between the adenoma and normal anterior pituitary, and found that it had a similar structure to the normal anterior lobe tissue with a thickness of about 0.5-1.0 mm, with less cells and more stroma (8). Faraoud et al. found that the pseudocapsule consisted of several enlarged cell-cords, which were surrounded by the basement membrane (13). However, it was only observed in a few cases, and it only existed in the margin of lesions and the tumor. In 2005, Kawamata et al. conducted histological studies of microsurgical pseudocapsules, and found that the pseudocapsules contained both normal pituitary cell layers and fibrous tissue, and it could be easily distinguished from the surrounding normal glands by the absence of connective tissue (9). In 2006, Oldfield et al. described the histological formation of the pseudocapsule in detail (10). In the early stage of PitNETs (<2 mm), the monoclonal proliferation of tumor cells began to compress the surrounding normal pituitary tissue without causing tissue displacement. As the adenoma enlarged or expanded (2-4 mm), the surrounding compressed normal pituitary tissue was displaced, and a relatively obvious membranous tissue surrounding the tumor was produced, forming the pseudocapsule. Further enlargement or expansion might invade or destroy the pseudocapsule and infiltrate the surrounding structures. Pathological studies confirmed that the pseudocapsule consisted of several compressed layers of individual acini and their reticulin envelope, a connective tissue identical to type III collagen, which played a pivotal role in maintaining the strength and integrity of the capsule. This theory was later widely accepted.

In 2009, by histopathological studies, Lee et al. demonstrated that the pseudocapsule was composed of fibroblasts, collagen, and small cell aggregates, which could become incomplete or discontinuous as the tumor enlarged; in some cases, tumor cell infiltration was present (11). In 2013, by endoscopic observation and histopathological examination, Ceylan et al. confirmed that the pituitary capsule and the pseudocapsule were two different structures, and the pseudocapsule was composed of multiple layers of compressed reticulin with collagen fibers, fibroblasts, and small cell clusters, but few connective tissues (18). In 2019, Nagata et al. reported histological evidence for the presence of pseudocapsules through neuroendoscopic resection of a portion of the thin normal anterior pituitary tissue for pathological examination in functional PitNETs lacking a clear pseudocapsule (19). It contains protein-like compact structure. In 2021, Zhou et al. reported that even no pseudocapsule was visualized with endoscopy, the evidence of a pseudocapsule was found by histopathology in adjacent normal pituitary tissue, composed of fibroblasts, collagen fibers, and small cell clusters (20). It is generally acknowledged that the pseudocapsule originated between the tumor and the anterior pituitary lobe. Weil et al. found that PitNETs in the posterior pituitary lacked a reticular fibrous structure, thus indirectly confirming that the pseudocapsule was mainly derived from the anterior pituitary (21). However, Taylor et al. believed that the pseudocapsule was located between the tumor and the posterior pituitary lobe, and the pseudocapsule could be stripped along the margin of the posterior lobe (22).

### Morphological characteristics

The pseudocapsule is different from normal pituitary tissue and adenoma in appearance and texture. The normal pituitary gland is pale yellow or orange colored while the pseudocapsule is often grayish-white. Also, the adenoma is soft in texture, and the texture of the pseudocapsule is typically tough. The thickness of the pseudocapsule is 0.5-1.0 mm in general, and it can evolve into the following forms as the tumor grows (11): 1. well-developed capsule that completely covers the tumor; 2. thin, fibrous capsule; 3. thick fibrous tissue capsule; 4. pale yellow, normal gland-like, thin capsule; 5. calcified and dense fibrolated capsule. It has been reported that the pseudocapsule could also appear yellowish in color or transparent during surgeries, with a tough texture (23, 24). The surface can be smooth or adhesive and difficult to be peeled off, especially when it is adhered to the arachnoid membrane or the inner wall of the cavernous sinus. For smaller adenomas, the pseudocapsule can be well-developed and completely wrap the tumor; for larger adenomas, the pseudocapsule may become irregular or incomplete once its integrity is destroyed. The tumor cells thus infiltrate the pseudocapsule to invade the adjacent structures such as the surrounding normal tissue or the cavernous sinus. It has been reported that approximately 50 percent of the pseudocapsules contain infiltrated tumor cells (11, 16, 25).

## Relationship between the pseudocapsule and PitNETs

### Relationship with the size of PitNETs

The pseudocapsule is related to the tumor size during its formation and development. When the adenoma is small (microadenoma), the pseudocapsule is only partially formed in the stage of incomplete development. As tumor enlarged in size (macroadenoma), the pseudocapsule is well developed and easily identified. When the tumor grows further (giant adenomas), the fibrous degeneration caused by compressing the pseudocapsule results in rupture or infiltration, which may cause the pseudocapsule to be incomplete and difficult to be recognized. The pseudocapsules are easier to be detected in adenomas of 2-3mm in sizes as proposed by Oldfield (10). Therefore, the incidence of pseudocapsules is related to adenoma size, but not positively related. It is believed that about 50% of PitNETs are found to have a complete pseudocapsule (9, 11, 15). The pseudocapsule incidence in microadenomas, macroadenoma, and giant adenomas has been reported to be 22.33%, 75.73%, and 1.94%, respectively (26). However, some believe that even as the tumor enlarged and infiltrated the surrounding structure, a complete pseudocapsule could still be formed by the tumor caused compression (16). In recent years, with neuroendoscopy assisted PitNET resection, a complete pseudocapsule is more likely to be found in small adenomas than the larger ones, thus suggesting that the pseudocapsule is related to the size of the tumor. The presence of a complete pseudocapsule makes it easier to perform complete resection along the pseudocapsule. For recurrent tumors, the pseudocapsule is usually difficult to be visualized or even absent.

### Relationship with the endocrine function of PitNETs

The presence of a pseudocapsule has been reported to be associated with the endocrine functions of PitNETs (12). The incidence of pseudocapsules is different in functional and non-functional adenomas. The incidence also varies depending on the hormone secretion types of functional adenomas. Lee et al. found that in 616 patients, only 55.7% of PitNETs were found to have a pseudocapsule, and the incidence of pseudocapsules in NFPA, PRL, GH and ACTH adenomas was 50.7%, 70.9%, 55.0%, and 40.0%, respectively (11). Wang et al. analyzed 254 PitNETs, and found that the incidence of pseudocapsule in NFPA, PRL, GH and ACTH adenomas was 50.0%, 65.7%, 55.3%, and 36.4%, respectively (27). Among them, the incidence of pseudocapsule in PRL and GH adenomas are more frequent, which may be related to the fibrotic hyperplasiause promoted by the use of dopamine receptor agonists in PRL adenomas and the growth hormone secreted by GH adenomas (14, 15). The drug-promoting pseudocapsules are found to be more adhesive during operations and harder to be removed.

### Relationship with the biological behavior of PitNETs

The expansion of PitNETs may infiltrate the pseudocapsule or even the dura mater, irrespective of tumor types. Tumor cell infiltration into the pseudocapsule has been reported (11, 12, 15). PitNETs can be divided into invasive and non-invasive adenomas according to their growth pattern. Hardy grading is used for imaging diagnosis of the invasiveness of PitNETs. Lee et al. found that the incidence of pseudocapsules was 57.5%, 61.9%, and 53.6% in tumors of Hardy Types I, II, and III, respectively (11). The tumor infiltration into the pseudocapsule was reported in 28.6%, 43.3%, and 46.4% of the Hardy Types I, II, and III tumors, respectively. Zhang et al. reported that 47.72% invasive adenomas and 57.28% non-invasive adenomas were wrapped by pseudocapsules (26). Wang et al. reported that the pseudocapsules were recognized in 22.6%, 35.3%, and 42.1% of the tumors of Hardy Types I, II, and III, respectively (27). While these datas vary, the pseudocapsules are more common in the tumors of Hardy Types II and III than those of Hardy Types I and IV in general. This is probably due to the fact that the tumors of Hardy II and III are wrapped in relatively complete pseudocapsules, thus being more distinguishable. Hardy I tumors are less invasive, so the pseudocapsules are somehow not prominent. When the tumors grow into Hardy IV, the pseudocapsules can be incomplete or even missing.

### Relationship with tumor apoplexy of PitNETs

Lee et al. found that pituitary apoplexy affected the color and texture of the pseudocapsule (11). Sugawara et al. suggested that the sudden increase in tumor size at pituitary apoplexy promoted the formation of pseudocapsules (28). Li et al. reported that pseudocapsules were found in 7 cases of cyst degeneration after pituitary apoplexy (29). Pituitary apoplexy was assumed to enlarge the tumors by squeezing normal pituitary tissue, thus promoting the formation of pseudocapsules.

## Significance of the pseudocapsule in PitNETs resection

With the concept of pseudocapsule, the surgical approaches of PitNETs have progressed from the removal of the adenoma and the covering of the adenoma (including the pituitary capsule, the pseudocapsule, and the dura mater, initially developed by Wrightson) (8) to the dissection between the adenoma and the pituitary capsule, removing the adenoma and pituitary capsule while keeping the dura mater, as proposed by Chucko et al. (30), and then followed recently by the dissection along the interface between the adenoma and the normal pituitary tissue to entirely remove both the adenoma and the pseudocapsule, in order to achieve complete tumor resection and preserve normal pituitary function, which is known as the technique of ER, as proposed by Kawamata (9), Oldfield (10), Lee (11), and Jagannathan (16). Qu X et al. described in their review that some proposed an “extended resection” method for ACTH adenomas, that is, to remove the additional tissue about 1.5mm in thickness around the adenoma, to achieve complete tumor resection and biochemical remission. This thickness is indeed equivalent to the thickness of the pseudocapsule (12). Oldfield et al. described the surgical technique of ER in detail with surgical illustrations, and proposed that for small adenomas, the adenoma and pseudocapsule can be removed together along the capsular separation (10). For larger tumors, the tumor can be decompressed by IR first, and then separated along the pseudocapsule for complete resection. This approach is increasingly accepted by neurosurgeons in PitNET surgeries. Overall, with the widespread use of neuroendoscopy in PitNET resection, ER has been widely used for PitNETs of different types, sizes, and ages, and achieved good surgical results (14, 18, 25, 31–33).

Based on whether the pseudocapsule was removed during the operation, the surgical methods can be distinguished between ER and IR (which is also known as “subpseudocapsular resection” or “conventional resection”) in PitNETs (**Figure 1**). Recent studies (14, 15, 20, 32, 34) have reported that ER is a more effective surgical approach comparing with IR, with maximized GTR, less tumor remnants and recurrences, and higher endocrine remission rates. The ER technique is performed along the anatomical interface between the tumor and the normal pituitary tissue, with more advantages in the resection of PitNETs, including helping to distinguish microadenomas from normal pituitary tissue, especially MRI-negative ACTH adenomas, thus reducing the misresection of normal pituitary tissues (16); providing a protective layer to prevent the risk of cerebrospinal fluid leakage (CSF) from entering the subarachnoid space (10, 12); identifying the gap where the tumor breaks through the pseudocapsule and the tumor remnants for invasive PitNETs, which helps to guide radiation therapy after the surgery (16, 35). However, there are also some disadvantages in ER, such as repeated stretching maneuvers and excessive pursuit of pseudocapsular resection, which theoretically may increase the risk of CSF leakage and hypopituitarism, although currently no evidence supporting this potential downside has been reported.

## ER and the prognosis of PitNETs

### ER and the rate of GTR, biochemical remission, recurrence

The recurrence and incomplete biochemical remission of PitNETs are often caused by incomplete tumor resection. Literature reports have confirmed the presence of tumor cell infiltration within the pseudocapsule of PitNETs, and any residual, small fragmented capsule may lead to tumor recurrence and low biochemical remission rates, especially in ACTH and GH adenomas (11, 15, 22, 34). Lee et al. performed enlarged resection of the microsurgical pseudocapsule on 616 patients with PitNETs (11). The surgical remission rate with and without a pseudocapsule presence is 86.2% and 94.3%, respectively. Recurrence rate of tumors after total resection and subtotal resection are 0.8% and 42.1%, respectively. The results suggest that any tumor remnant in the pseudocapsule may lead to recurrence, which is also an obstacle to achieving complete biochemical remission. Pseudocapsule resection can help improve the rate of GTR and biochemical remission. In the analysis of 483 cases of ACTH-secreting adenomas by Jagannathan et al. (16), the biochemical remission rate of 261 cases of ER was almost 100% (one of them died of cirrhosis during follow-up), and the recurrence rate was only 2.3% (6/261), indicating that pseudocapsule exploration is an effective method to accurately identify adenomas in surgery for MRI-negative ACTH adenomas.

We reviewed the relevant literatures on the use of ER and IR in PitNET surgery in the past 10 years by neuroendoscopy or microscopy with neuroendoscopy-assisted transsphenoidal surgery (14, 15, 20, 22, 32–34, 36–39) (**Table 1**). We found that the GTR rate, biochemical remission rate, and tumor recurrence rate of ER were 83.7%-100%, 76.7%-93.5%, 1.6%-3.1%, respectively; while the same rates of IR were 54.5%-100%, 42.9%-85.3%, 2.4%-14.06%, respectively. Almost all these publications indicated that ER was superior to IR in terms of GTR rate, biochemical remission rate, and tumor recurrence rate, with statistical significance (P<0.05) (**Table 1**). Zhang X et al. (40) conducted a meta-analysis of ER and IR surgical resection of PitNETs, and a total of 1768 patients were included in the analysis (647 ER cases and 1121 IR cases). After sensitivity and heterogeneity analysis, ER was demonstrated to be superior to IR in the terms of the GTR and biochemical remission. Kinoshita et al. (33) found that no data indicated that ER was associated with greater risk in elderly patients by TNTS and there was no significant difference in the surgical outcomes and complications between the elderly (≥70 years old) and adults (<70 years old) patients, suggesting that ER is also safe and effective in non-invasive surgery for functional PitNETs in the elderly.

**Table 1:** ER and the rate of the GTR, Biochemical/Endocrinological remission, Tumor recurrence.

Author	Year	Sampal size		GTR			Biochemical/Endocrinological remission			Tumor recurrence		
		ER	IR	ER	IR	*P*	ER	IR	*P*	ER	IR	*P*
Qu X (34)	2011	78	64	71(91%)	44(68.8%)	0.002	72(92.3%)	45(70.3%)	0.001	2(2.56%)	9(14.06%)	0.023
Kim EH (15)	2015	263	826	258(98.1%)	733(93.6%)	0.004	129(90.2%)	302(85.3%)	0.145	8/258(3.1%)	8/164(4.9%)	0.055
Xie T (14)	2016	21	22	18(85.7%)	12(54.5%)	0.025	18(85.7%)	12(54.5%)	0.028	–	–	-
Liu T (36)	2017	157	130	141(89.8%)	106(81.5%)	0.044	50/58(86.2%)	26/46(56.5%)	0.001	3(1.9%)	9(6.9%)	0.035
Xu Q (38)	2018	22	21	21(95.5%)	15(71.4%)	0.046	10/11(90.9%)	3/7(42.9%)	0.047	–	–	-
Taylor DG (22)	2018	74	34	49(66.2%)	15(44.1%)	-	–	–	-	–	–	-
Xie Zh (37)	2019	75	58	75(100%)	58(100%)	-	70(93.3%)	41(70.7%)	<0.001	–	–	-
Li QX (32)	2019	116	90	97(83.7%)	62(68.9%)	0.028	89(76.7%)	53(58.9%)	0.006	–	–	-
Wang X (39)	2020	55	63	49(89.1%)	49(77.8%)	0.036	48(87.3%)	35(55.6%)	0.000	–	–	
Kinoshita Y (33)^a^	2021	92	114	86/92(93%)	87/114(76%)	0.001	–	–	-	–	–	-
Kinoshita Y (33)^b^	2021	21	45	21(100%)	33(93%)	0.0068	–	–	-	–	–	-
Zhou Y (20)	2021	64	125	62(96.9%)	107(85.6%)	0.0171	29/41(93.5%)	40/53(75.5%)	0.0369	1(1.6%)	3(2.4%)	0.705

^a^(younger <70 years); ^b^(elderly ≥70 years).

In conclusion, we believe that comparing with IR, ER can improve the rate of GTR and biochemical remission while lowering the recurrence. With the application of neuroendoscopy technology, we can identify the pseudocapsule more clearly, the external resection along the pseudocapsule can better protect the functions of normal pituitary gland, the GTR and biochemical remission rate was improve to 89.31% and 86.34%, and the recurrence rate was reduced to almost zero (40). We would like to recommend that the pseudocapsules be removed as aggressively as possible in order to reduce tumor remnants and recurrences, and improve endocrine remission rate, especially for some functional adenomas such as ACTH and GH-secreting adenomas, as well as the high-risk PitNETs as described in the 2017 WHO classification of endocrine organs tumors, including sparsely granulated growth hormone adenomas, male lactation for steroid adenomas, Crooke cell adenomas, and corticotropin-silencing adenomas (41).

### ER and postoperative complications

Comparing to IR, ER requires more aggressive peeling from the lower pituitary or the arachnoid layer. Sometimes the capsule is tightly adhered to the arachnoid membrane of the septum sellea, so that pursuing total excision of the capsule, repeated traction, and violent manipulation may theoretically increase the risk of CSF leakage and damage the normal pituitary gland or pituitary stalk. Postoperative CSF leakage is the most frequent complication of the transsphenoidal surgery, with an incidence of 0.5%-6.0% (15). However, with the continuous improvement of neuroendoscopy techniques and sellar bottom reconstruction techniques, the incidence of CSF leakage has been greatly reduced.

We reviewed the postoperative complications reported in the above 11 literatures (**Table 2**). The incidence rates of intraoperative CSF leakage, postoperative CSF leakage, transient diabetes insipidus, and hypopituitarism in ER were 22.7%-59.3%, 0-22%, 0-73.3%, 4.8%-26.7%, respectively; and the same rates in IR were 3.4%-41.6%, 0-38%, 2%-69%, 4.5%-17.2%, respectively. Although few literatures suggest that IR is better than ER in intraoperative CSF leakage (15, 20, 32, 38), almost all literatures confirm that there is no statistical difference in the incidence rates of postoperative CSF leakage, transient diabetes insipidus, and hypopituitarism (P>0.05) (**Table 2**). A meta-analysis conducted by Zhang X et al. (40) also found that there was no statistically significant difference between ER and IR in the incidence of intraoperative CSF leakage, postoperative CSF leakage, diabetes insipidus, and hypopituitarism. Therefore, we have reasoned that comparing to IR, ER does not increase the risk of postoperative complications. It also should be pointed out that intraoperative CSF leakage may not necessarily lead to postoperative CSF leakage. Intraoperative CSF leakage can be repaired by using materials such as autologous fat and fascia, artificial biofilms, mucosal flaps, biological protein glue, and sutures and materials of the sellar bottom dura. In addition, postoperative indwelling lumbar drainage system could reduce the incidence of postoperative CSF leakage. The incidence of postoperative CSF leakage will be lower with the continuous improvement of neuroendoscopy equipments and techniques, as well as the improvement of sellar bottom reconstruction techniques.

**Table 2:** ER and the complications after operations,such as Intraoperative CSF leakage,Postoperative CSF leakage,Transient and Permanent DI,Hypopituitarism.

Author	Year	Sampal size		Intraoperative CSF leakage			Postoperative CSF leakage			Transient DI			Permanent DI			Hypopituitarism		
		ER	IR	ER	IR	*P*	ER	IR	*P*	ER	IR	*P*	ER	IR	*P*	ER	IR	*P*
Qu X (34)	2011	78	64	–	–	-	6(7.7%)	2(3.1%)	0.295	21(26.9%)	16(25%)	0.795	–	–	–	7(11.1%)	4(7.3%)	0.474
Kim EH (15)	2015	263	826	156(59.3%)	344(41.6%)	<0.001	11(4.2%)	22(2.7%)	0.211	–	–	-	–	–	–	36/235(15.3%)	82/723(11.3%)	0.127
Xie T (14)	2016	21	22	8(38.1%)	8(36.4%)	0.646	4(19%)	2(9.1%)	0.646	3(14.3%)	6(27.2%)	0.457	–	–	–	1(4.8%)	3(13.6%)	0.607
Liu T (36)	2017	157	130	52(33.1%)	45(34.6%)	0.790	24(2.5%)	3(2.3%)	1.000	19(12.1%)	17(13.1%)	0.804	–	–	–	12(7.6%)	9(6.9%)	0.816
Xu Q (38)	2018	22	21	10(45.6%)	3(14.3%)	0.045	1(4.5%)	1(4.8%)	1.000	7(3.2%)	7(3.3%)	0.537	–	–	–	–	–	-
Taylor DG (22)	2018	74	34	–	–	-	16(22%)	13(38%)	0.069	–	–	-	–	–	–	–	–	-
Xie Zh (37)	2019	75	58	17(22.7%)	6(10.3%)	-	–	–	-	49(65.3%)	40(69%)	0.659	–	–	–	20(26.7%)	10(17.2%)	0.197
Li QX (32)	2019	116	90	31(26.7%)	12(13.3%)	0.019	2(1.7%)	1(1.1%)	1	85(73.3%)	57(63.3%)	0.126	–	–	–	–	–	-
Wang X (39)	2020	55	63	14(25.5%)	13(20.6%)	0.534	2(3.6%)	4(6.3%)	0.503	13(23.6%)	16(25.4%)	0.825	1(1.8%)	2(3.2%)	0.641	–	–	-
Kinoshita Y (33)^a^	2021	92	114	33(36%)	38(33%)	0.7686	0	1(0.9%)	1.0	3(14%)	7(16%)	1.000	1(4.8%)	0	0.3182	–	–	-
Kinoshita Y (33)^b^	2021	21	45	8(38%)	11(24%)	0.3815	0	0	1	18(20%)	29(25%)	0.404	0	3(2.6%)	0.2529	–	–	-
Zhou Y (20)	2021	64	125	18(28.1%)	17(13.6%)	0.015	0	1(1.6%)	0.4731	31(48.4%)	80(64%)	0.0397	–	–	–	–	–	-

^a^(younger <70 years); ^b^(elderly ≥70 years).

Although the previous literature reported that ER resulted in better outcomes in PitNETs surgeries, some argue that the pituitary function conservation is more important than the total removal of PitNETs and psudocapsules; and even if the tumor recurs, repeated surgery is safe with experienced surgeons and can be tolerated by most patients (42). It is possible that sometimes a small amount of pseudocapsule is closely adhered to the thin arachnoid membrane of the septum sella, and an attempt to completely remove the pseudocapsule may lead to catastrophic CSF leakage. Whether the little residual pseudocapsule of PitNETs will definitely cause recurrence, especially for non-functional PitNETs, is also a subject for debating. Some believe that the recurrence of PitNETs depends mainly on the tumor cell infiltration of the cavernous sinuses rather than the pseudocapsule (25). In our clinical experience, we conducted pathological examination on the medial wall of cavernous sinus of some functional PitNETs, and found that there was tumor cell infiltration, which might also explain the reason for some tumor recurrence. Therefore, we think that the heterogeneity of PitNETs should be fully considered and an individualized surgical protocol is needed for each patient. If the total resection of tumor might cause severe adverse effects, it should be avoided. Follow-up treatment such as radiotherapy maybe used for difficult cases.

## Conclusions

It has been agreed that the pseudocapsule is composed of multiple layers of reticular protein compression, containing fibroblasts, collagen fibers, and small cell clusters, with few connective tissue components. Tumor cell infiltration can be observed in some cases. The presence of residual tumor cells in pseudocapsule is a potential source of tumor recurrence and incomplete endocrine remission. ER can be applied in the operation of PitNET patients of different types, sizes, and ages. The presence of pseudocapsule helps to identify the PitNETs and normal pituitary tissues during the surgeries. When the tumor is small or the pseudocapsule is well developed, the capsule can be used as a surgical anatomical interface, along which the tumor and pseudocapsule can be completely removed. When the tumor is large or the pseudocapsule is incomplete, the tumor can be decompressed firstly through IR, then the pseudocapsule can be found and peeled off as much as possible, and any suspected pseudocapsule fragment could be completely removed. Endoscopic-assisted ER can improve the rate of GTR and biochemical remission, and reduce the rate of tumor recurrence, and will not increase the risk of postoperatively complications.

## Author contributions

Conception and design of the review: ZW and XZ. Drafting the manuscript and the figure: XB. Modifying the manuscript critically for important content: LX, TH, JM and CH. All authors contributed to the article and approved the submitted version.

## Funding

This work was supported by the National Natural Science Foundation of China under Grant Number 82141114, 81972339 (ZBW), Shanghai Municipal Science and Technology Commission under Grant Number 18XD1403400 (ZBW), Qiusuo Funds for Distinguished Young Scholar (LX), Medical Science and Technology Project of Henan Province (joint construction) LHGJ20190508 (XBW).

## Acknowledgments

We thank Ke Yang for drawing the **Figure 1**.

## Conflict of interest

The authors declare that the research was conducted in the absence of any commercial or financial relationships that could be construed as a potential conflict of interest.

## Publisher’s note

All claims expressed in this article are solely those of the authors and do not necessarily represent those of their affiliated organizations, or those of the publisher, the editors and the reviewers. Any product that may be evaluated in this article, or claim that may be made by its manufacturer, is not guaranteed or endorsed by the publisher.
